# Chronic Disseminated Candidiasis Complicated by Immune Reconstitution Inflammatory Syndrome in Child with Acute Lymphoblastic Leukemia

**DOI:** 10.1155/2016/5960150

**Published:** 2016-10-09

**Authors:** Olga Zając-Spychała, Bogna Ukielska, Katarzyna Jończyk-Potoczna, Benigna Konatkowska, Jacek Wachowiak

**Affiliations:** ^1^Department of Pediatric Oncology, Hematology and Transplantology, University of Medical Sciences, Poznan, Poland; ^2^Department of Pediatric Radiology, University of Medical Sciences, Poznan, Poland

## Abstract

Hepatosplenic candidiasis also known as chronic disseminated candidiasis is a rare manifestation of invasive fungal infection typically observed in patients with acute leukemia in prolonged, deep neutropenia. Immune reconstitution inflammatory syndrome (IRIS) is an inflammatory disorder triggered by rapid resolution of neutropenia. Diagnosis and treatment of IRIS are still challenging due to a variety of clinical symptoms, lack of certain diagnostic criteria, and no standards of treatment. The diagnosis of IRIS is even more difficult in patients with hematological malignancies complicated by “probable” invasive fungal infection, when fungal pathogen is still uncertain. We report a case of probable hepatic candidiasis in 4-year-old boy with acute lymphoblastic leukemia. Despite proper antifungal therapy, there was no clinical and radiological improvement, so diagnosis of* Candida*-related IRIS was made and corticosteroid therapy was added to antifungal treatment achieving prompt resolution of infection symptoms.

## 1. Introduction

Chronic disseminated candidiasis (CDC) also known as hepatosplenic candidiasis (HSC) is a severe form of disseminated fungal infection affecting mainly the liver, the spleen, and occasionally the kidneys [1]. The incidence of CDC increases parallel to increasing usage of aggressive chemotherapy causing prolonged deep neutropenia and is estimated at <5% of patients treated for acute leukemia [2, 3]. CDC remains an important cause of treatment-related mortality in leukemia patients [4].

The immune reconstitution inflammatory syndrome (IRIS) initially described in HIV infected patients during antiretroviral therapy is a phenomenon of clinical deterioration occurring during immune recovery. IRIS occurs as an exuberant inflammation resulting from “unmasking” of previously clinically silent infection at a time point of neutrophil recovery [5].

## 2. Case Report

**Figure 1:**
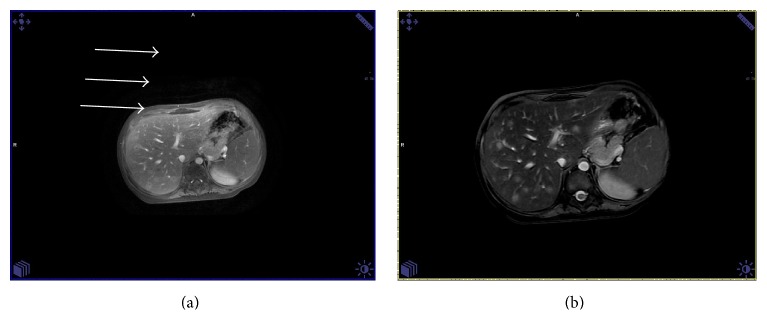
Focal liver lesions in MR imaging (hypointensive in T1-weighted contrast enhanced and fat saturated sequences (a); hyperintense in T2-weighted scans (b)).

**Figure 2:**
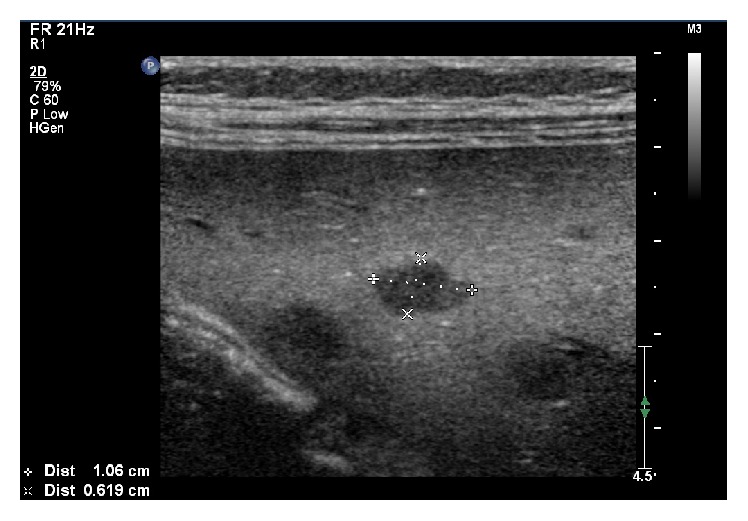
Fungal microabscesses in hepatic ultrasonography at diagnosis.

**Figure 3:**
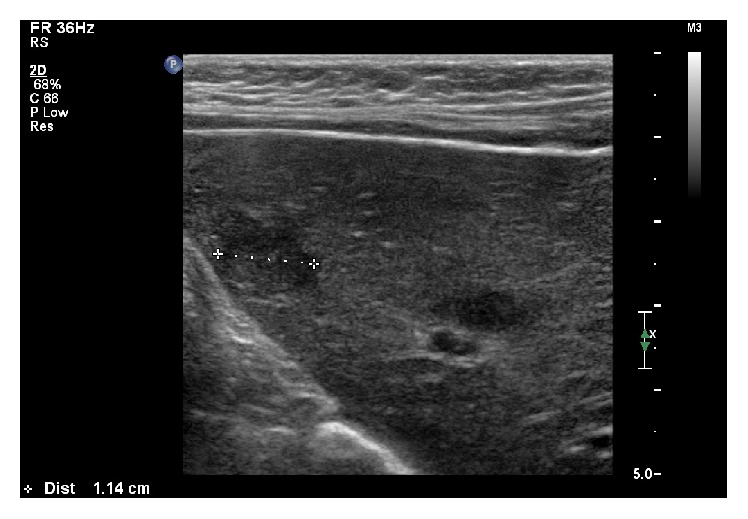
Liver microabscesses after 2 weeks of antifungal treatment.

A 4-year-old boy was diagnosed with common ALL and started treatment according to ALL IC-BFM 2009. Patient was stratified into high risk group, but there was remission achieved on time (day +33). Since the beginning of chemotherapy, routine antifungal prophylaxis of fluconazole was used. On day +35 of remission induction chemotherapy, he developed severe lower gastrointestinal tract (GI) hemorrhage, necessitating transfer to the intensive care unit. He received red blood cells, platelet, and plasma transfusions along with inotropic support and empirical antibiotics. Abdominal ultrasonography (US) and computed tomography (CT) scan were unremarkable. After initial stabilization, an urgent endoscopy revealed extensive mucosal ulceration involving the entire colon. Biopsy was not performed due to the risk of perforation. Complete blood count and coagulation profile were within normal ranges. Over next 24 hours, the hemorrhage was successfully stopped and the clinical status of patient became stable. However, 2 weeks later the patient developed febrile neutropenia refractory to empiric broad-spectrum antibiotic treatment (meropenem, metronidazole, and teicoplanin). Blood cultures, pharynx, and stool swabs were negative. Voriconazole (8 mg/kg twice a day) and then Amphotericin B (up to 5 mg/kg) were empirically added to antimicrobial treatment, but fever persisted. Based on abdominal magnetic resonance imaging (MRI) hepatic microabscesses (Figure 1) were diagnosed. They were also found in hepatic ultrasonography (Figure 2). A biopsy of these lesions under US was performed. The culture was negative and histopathological examination revealed only lymphocytes and neutrophils lesions, but no fungus. As mannan antigen was positive, according to European Organization for Research and Treatment of Cancer Mycosis Study Group, “probable” invasive fungal infection (IFI), that is, hepatic candidiasis, was diagnosed and we switched the treatment to caspofungin (50 mg/m^2^). The persistent fever and elevated inflammatory markers (C-reactive protein, CRP 10,34 mg/dL) despite good clinical condition, appropriate antifungal treatment, still negative blood cultures, and neutrophil recovery raised a suspicion of candidiasis-related IRIS and corticosteroids (dexamethasone 0,5 mg/kg) were added to caspofungin therapy. Fever disappeared after 3 days, CRP level was normalized within 10 days, and significant decrease in size and number of liver microabscesses after 2 weeks was achieved (Figure 3). Corticosteroid treatment was tapered in 7 days and then stopped. Caspofungin treatment was continued for 3 months together with continuation of chemotherapy. After significant regression of liver microabscesses we tried to switch into itraconazole, but radiological progression in US occurred. Finally, patient was treated for 6 months with caspofungin and, after the antifungal therapy completed, the MRI returned to normal with no pathological lesions in the liver. The patient is already in ALL remission for 25 months.

## 3. Discussion

Diagnostics and therapy of* Candida spp*. infections remain one of the most frequent complications in neutropenic patients. In addition to candidemia accompanied with continuously high mortality reaching up to 30%, CDC is also clinically relevant problem, especially for patients with hematological malignancies treated with aggressive chemotherapy regimens [6, 7].

The pathophysiology of CDC is not fully understood; however its clinical and radiological features have been characterized. It is assumed that initial colonization of the gastrointestinal tract with* Candida* species, followed by liver invasion through portal venous circulation, is the most probable cause of the disease [8]. The course of CDC has two clinical stages: first phase with fungal dissemination during aplasia of the patient, especially when coinciding with the mucositis or ulceration along with the gastrointestinal tract damaged by chemotherapy, leading to translocation of yeasts colonizing the colon into the bloodstream and the other stage when hematological restoration together with “paradoxical” clinical deterioration (recurring fever and the appearance of hepatic micronodules) occurs. This phase is probably caused by recovery of pathogen-specific immunity characterized by proinflammatory Th1 and Th17 response and production of tumor necrosis factor-*α* (TNF-*α*) and interferon *δ* (IFN-*δ*). The antifungal response rapidly becomes excessive with immunological Th1/Th17–Th2/Treg imbalance, responsible for the formulation of granulomatous lesions in tissue containing the pathogen. The local immunological dysregulation promotes the immune reconstitution inflammatory syndromes (IRIS) occurrence [3, 8]. It is also reasonable that there is the corticosteroids spectacular response by inhibiting secretion of proinflammatory cytokines and stimulation of the anti-inflammatory ones. In our patient, the first phase of CDC was due to massive ulceration of the colon responsible for hemorrhage as well. As GI bleeding resulted in delaying ALL chemotherapy and patient had achieved remission, rapid hematological recovery promoted the second phase of CDC with immunological imbalance in favor of proinflammatory cytokines.

IRIS is a phenomenon defined as the appearance of inflammatory disorders and clinical worsening in patients treated due to opportunistic infection that cannot be explained by reinfection [9]. Better understanding of CDC etiology leads to hypothesis that it belongs to the spectrum of fungus-related IRIS [3]. As data of HIV infected patients report, the most common pathogen of fungus-related IRIS remains not* Candida* spp. but* Cryptococcus neoformans* caused severe meningitis [10]. Because of wide spectrum of IRIS clinical symptoms, diagnosis of this syndrome is problematic. Moreover, there is no available diagnostic test to diagnose the syndrome [11]. Until better understanding and establishing management of IRIS, the diagnosis is only clinically based and requires experience and vigilance of physicians.

From IRIS point of view, the essential and unsolved problem is time of effective antifungal treatment of CDC and the optimal delay in anticancer therapy. As IRIS patients are usually in remission and successful treatment of candidiasis requires several months of antifungal therapy, the consolidation introduction is extremely important time point [12]. Amphotericin B has been the cornerstone of treatment of severe fungal infections including CDC [13]. However, since prolonged treatment is necessary the great problem is toxicity, especially nephrotoxicity being the major complication that warrants discontinuation of Amphotericin. B. The newer options for candidiasis treatment are echinocandins characterized by better efficacy and toxicity profile in comparison with Amphotericin B in different setting of patients [14]. Our patient was successfully treated for CDC with caspofungin, but our greatest challenge was continuing cytotoxic agents simultaneously with antifungal therapy to achieve both remission of ALL and control of infection. The literature provides that successful treatment of CDC in patients with ALL does not need to interrupt chemotherapy cycles [12, 15]. The reported patient also safely continued both anticancer and antifungal treatment maintaining leukemia and candidiasis remission.

The other problem that need to be considered is the antileukemic effect of inflammatory reaction itself associated with a lower leukemia relapse rate [16]. On the other hand, the CDC is the important cause of mortality for patient treated for leukemia. Consequently, the antileukemic effect associated with inflammatory is counterbalanced by increased infection-related mortality, leading to a generally worse prognosis in patients who develop CDC. Therefore, it is critical to control CDC complicated by IRIS in patients with leukemia considering the fact that in the same way we suppress the antileukemic effect.

Summarizing, CDC remains severe complication occurring in leukemia patients and can be responsible for induction of the immune reconstitution inflammatory syndrome. Treatment of* Candida*-related IRIS requires both prolonged antifungal and corticosteroid treatment. Nevertheless, prognosis of patients with CDC is directly related to underlying leukemia. Recommendations concerning treatment of HSCT should be part of antifungal therapy guidelines, especially in patients treated with chemotherapy.
